# Trends in emergency department visits and hospitalization rates for inflammatory bowel disease in the era of biologics

**DOI:** 10.1371/journal.pone.0210703

**Published:** 2019-01-16

**Authors:** Gunn Huh, Hyuk Yoon, Yoon Jin Choi, Cheol Min Shin, Young Soo Park, Nayoung Kim, Dong Ho Lee, Joo Sung Kim

**Affiliations:** 1 Department of Internal Medicine, Seoul National University College of Medicine, Seoul, Korea; 2 Department of Internal Medicine, Seoul National University Bundang Hospital, Seongnam, Korea; University of Colorado, UNITED STATES

## Abstract

**Background:**

The use of biologics in inflammatory bowel disease (IBD) has increased recently. However, studies on whether the proportion of IBD patient visits to the emergency department (ED) has decreased are scarce. We investigated the trends in IBD-related ED visits and hospitalization rates.

**Methods:**

Medical records of IBD-related visits to the ambulatory department (AD) and the ED of the Seoul National University Bundang Hospital in 2007, 2009, 2012, and 2014 were reviewed. Multiple-variable logistic regression analysis was used to identify significant risk factors for hospitalization.

**Results:**

The proportion of IBD patients who visited ED was 12.3% in 2007, 9.7% in 2009, 8.3% in 2012, and 6.4% in 2014 (*P* = 0.002). The most common chief complaints were abdominal pain (66.9%) in Crohn’s disease (CD) patients and hematochezia (36.5%) in ulcerative colitis (UC) patients. The hospitalization rate following ED visits was 47.2% in CD patients and 55.6% in UC patients (*P* = 0.100). Multiple-variable analysis showed that significant risk factors associated with hospitalization in CD were aggressive disease behavior (odds ratio[OR] 3.54, *P* = 0.017) and presence of steroid exposure (OR 2.35, *P* = 0.047). Elevated C-reactive protein (CRP) (>0.5 mg/dL) (OR 5.40, *P* = 0.016) was the only risk factor associated with hospitalization in UC.

**Conclusions:**

The proportion of ED visits decreased from 2007 to 2014; there was no significant change in hospitalization rates. Disease behavior/presence of steroid exposure and elevated CRP were associated with hospitalization among CD and UC patients who visited the ED, respectively.

## Introduction

Inflammatory bowel disease (IBD), which consists of Crohn’s disease (CD) and ulcerative colitis (UC), is a chronic intestinal inflammatory disorder. In recent years, the incidence and prevalence of IBD has been increasing worldwide, resulting in a tremendous burden on healthcare resources.[1–3] The annual incidence rates vary by geographic region and are steadily rising in Asia. The mean annual incidence of UC in South Korea is 4.6 per 100,000 and that of CD is 3.2 per 100,000.[4, 5] A population-based study in South Korea including 236,106 patients with IBD showed that the overall annual healthcare costs for IBD had increased approximately two-fold from 2010 to 2014.[6]

The introduction of biologics since 2000 has improved quality of life of patients and has led to a steady decrease in hospitalization and surgery for IBD.[7–9] A meta-analysis reported that anti-TNF biologics reduced the odds of hospitalization for CD and UC by more than half and surgery by 33% to 77%.[10] However, studies on whether the proportion of ED visits by IBD patients has decreased are scarce. Previous studies on ED visits by IBD patients were based on data from the 1990s and early 2000s.[11, 12] An ED visit is an indicator that reflects acute disease flares or complications of IBD and is associated with quality of life in IBD patients. Therefore, it is important to understand the trends and patterns of ED visits, hospitalization rates, and factors associated with hospitalization, which thereby enable a better management of IBD patients in the ED.

The aim of this study was to investigate the trends in IBD-related ED visits and hospitalization rates. We also aimed to identify factors associated with hospitalization following ED visits in IBD patients.

## Materials and methods

### Study subjects

Medical records of IBD patients aged 17 or older who visited the ambulatory department (AD) and the ED at the Seoul National University Bundang Hospital in 2007, 2009, 2012, and 2014 were evaluated retrospectively. Diagnosis of CD or UC was confirmed by previously established international criteria based on clinical, endoscopic, histopathological, and radiological findings. Exclusion criteria included UC patients who had undergone total proctocolectomy with ileal pouch-anal anastomosis, patients who were first diagnosed with IBD on presentation to the ED, or patients who were transferred from another hospital. Proportions of the number of patients visiting ED compared to AD in each year were analyzed on a ‘per-patient’ basis. That is to say, repeated visits by the same patient were not counted in duplicate. We hypothesized that the number of patients visiting AD in a specific year could represent the number of patients treated in our hospital network. In analyzing patient characteristics and clinical outcomes at the ED, however, we performed a ‘per-visit’ analysis. In other words, each ED visit by the same patient was regarded as a different case.

Data regarding age, sex, type of IBD, disease duration, disease extent and behavior, time interval between the last AD visit and ED visit, current or past medications, and history of intestinal resection were collected. All patients were phenotyped using the Montreal classification.[13] The chief complaint of the ED visit; the Charlson comorbidity index[14]; serum levels of white blood cells (WBC), hemoglobin (Hb), C-reactive protein (CRP), and albumin; endoscopic or radiological evaluation; hospitalization duration; and surgical interventions were also evaluated. Reference values for WBC, Hb, CRP, and albumin levels were 4.0 x 10^3^/**μ**L to 10.0 x 10^3^/**μ**L, 12 to 16 g/dL, 0 to 0.5 mg/dL, and 3.3 to 5.2 g/dL, respectively.

### Statistical analysis

Continuous values are given as the mean±standard deviation and were compared using the independent t-test or the Mann-Whitney U test. Categorical values are presented as the number (percent) and were compared using chi-square tests or Fisher’s exact tests. Trends in ED proportions and hospitalization rates were evaluated by linear-by-linear association. Univariate logistic regression analysis was used to identify possible covariates as significant risk factors for hospitalization. Variables with *P* < 0.05 were then subjected to multiple-variable logistic regression analysis to identify independent contributors. All reported *P*-values are two-sided with a threshold of < 0.05 indicating significance. All statistical analyses were performed using the statistical software package SPSS 18.0 for Windows (SPSS Inc., Chicago, IL, USA).

### Ethical standards

This study protocol was approved by the Institutional Review Board of Seoul National University Bundang Hospital (B-1705-395-109). All data were encrypted using patient numbers as unique identifiers and the IRB waived the requirement for informed consent. The study was conducted in accordance with the Declaration of Helsinki.

## Results

### Trends in ED visits and hospitalization rates

**Fig 1:**
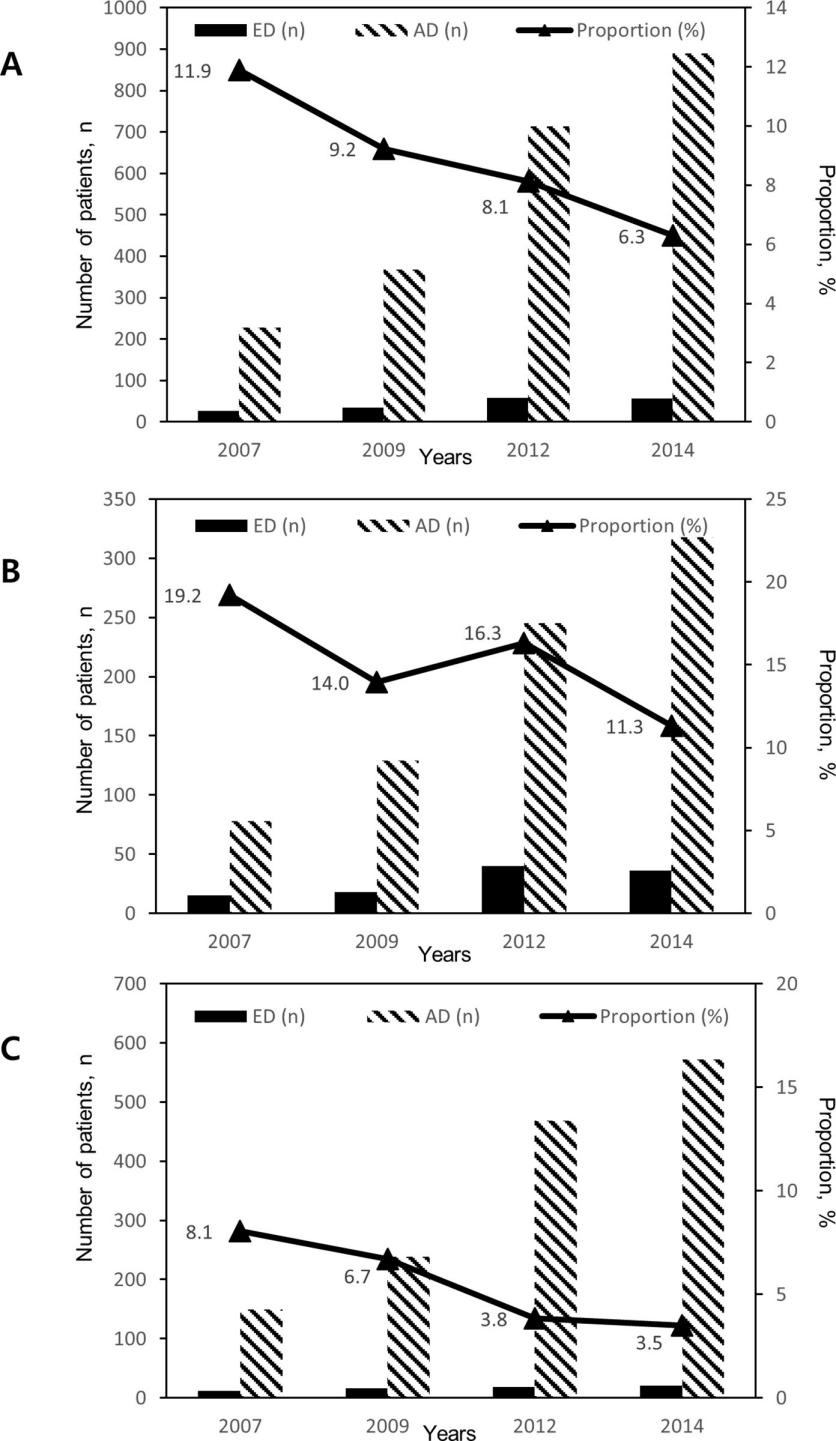
**The number and proportion of patients with IBD (A), CD (B), and UC (C) visiting ED and AD stratified by years.** The absolute number of IBD patients who visited ED increased but the ED proportion of total patients decreased from 2007 to 2014 (linear-by-linear *P* = 0.002).

The absolute number of IBD patients who visited ED increased, but the proportion of IBD patients visiting ED compared to AD decreased from 2007 to 2014 (11.9% in 2007, 9.2% in 2009, 8.1% in 2012, and 6.3% in 2014) (Fig 1). A linear trend was observed for the proportion of ED visits (*P* = 0.002). In CD patients, the proportion of patients visiting ED compared to AD was 19.2% in 2007, 14.0% in 2009, 16.3% in 2012, and 11.3% in 2014. (*P* = 0.081). In UC patients, the proportion of patients visiting ED compared to AD was 8.1% in 2007, 6.7% in 2009, 3.8% in 2012, and 3.5% in 2014. (*P* = 0.004). In general, the proportion of IBD-related ED visits was higher in CD patients than UC patients in each year.

**Fig 2:**
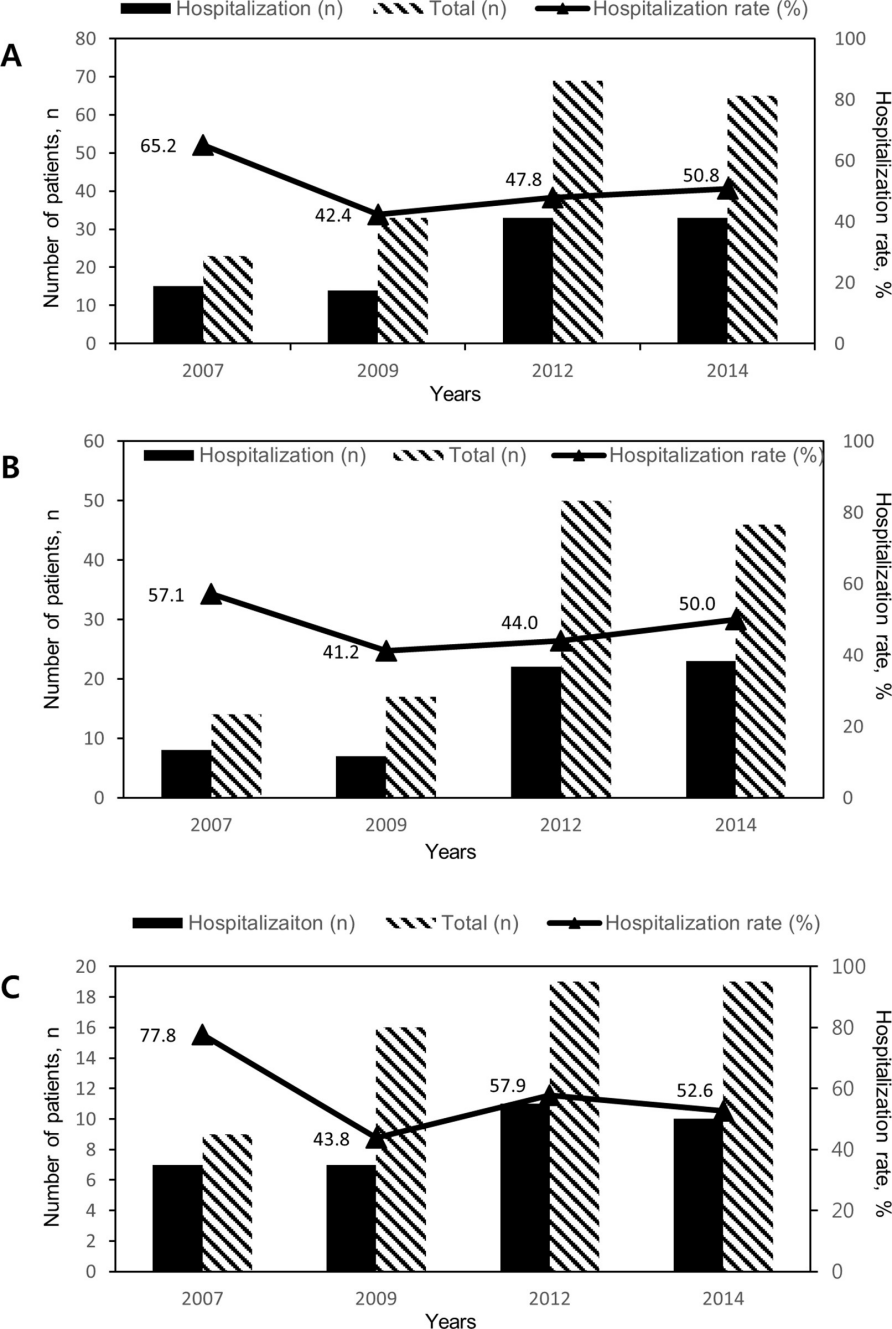
**Hospitalization rates of patients with IBD (A), CD (B), and UC (C) visiting ED stratified by years.** There was no significant linear trend for hospitalization rates of ED patients (*P* = 0.610).

There was no significant linear trend for hospitalization rates of ED patients (*P* = 0.610) (Fig 2). Hospitalization rate of IBD patients following ED was 65.2% in 2007, 42.4% in 2009, 47.1% in 2012, and 51.6% in 2014. In CD patients, hospitalization rate was 60.0% in 2007, 35.0% in 2009, 42.3% in 2012, and 50.0% in 2014 (*P* = 0.976). In UC patients, hospitalization rate was 75.0% in 2007, 53.8% in 2009, 61.1% in 2012, and 55.6% in 2014. (*P* = 0.528)

### Trends in the proportion of patients treated with biologics

**Fig 3:**
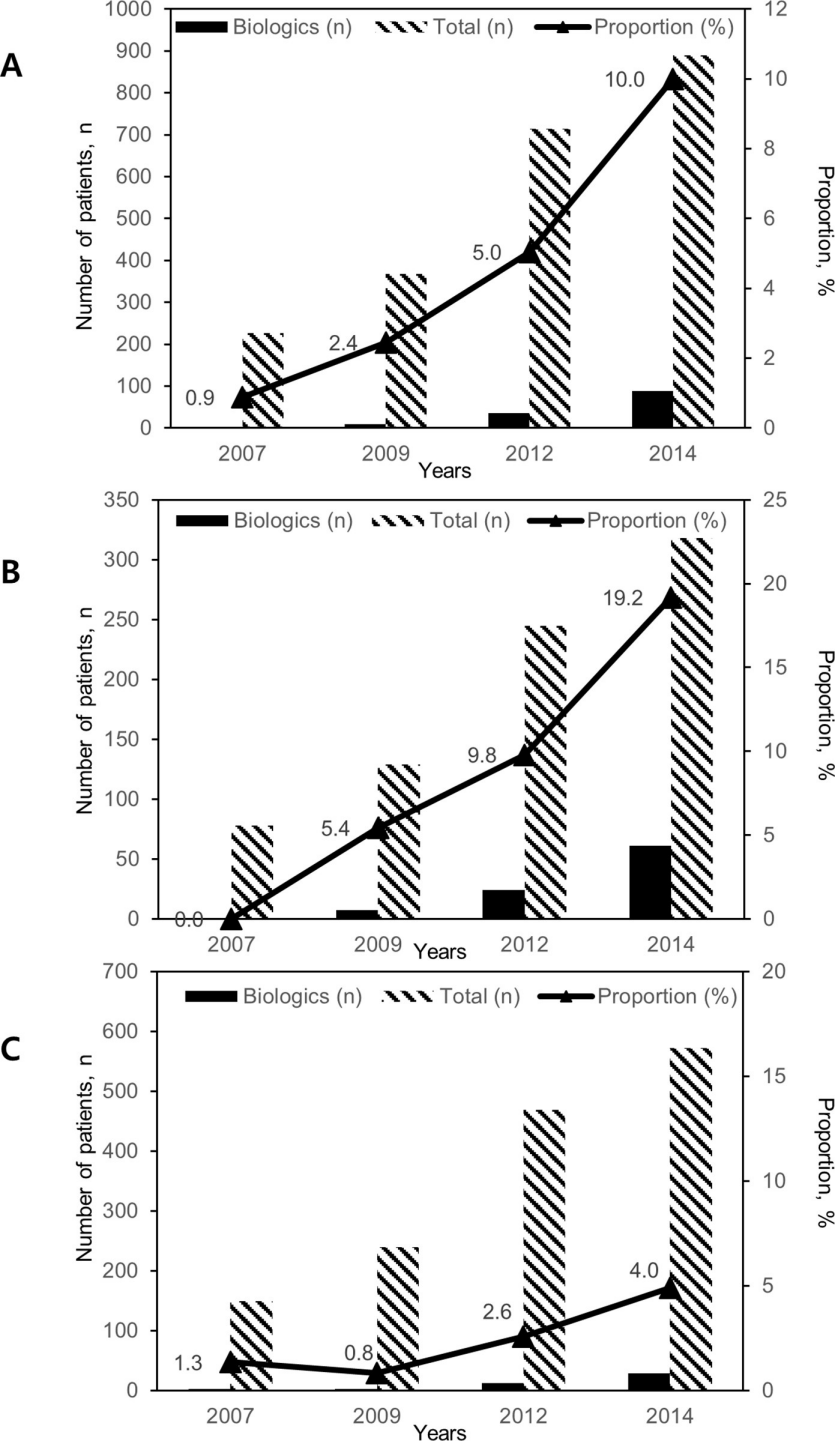
**The number and proportion of patients with IBD (A), CD (B), and UC (C) treated with biologics stratified by years.** The number and proportion of IBD patients who had received or under treatment with biologics increased from 2007 to 2014 (*P* <0.001).

The proportion of IBD patients who had received or under treatment with biologics increased from 2007 to 2014 (0.9% in 2007, 2.4% in 2009, 5.0% in 2012, and 10.0% in 2014) (Fig 3). A linear trend was observed for the proportion of patients treated with biologics (*P* <0.001). In CD patients, the proportion of patients treated with biologics was 0% in 2007, 5.4% in 2009, 9.8% in 2012, and 19.2% in 2014 (*P* <0.001). In UC patients, the proportion of patients treated with biologics was 1.3% in 2007 0.8% in 2009, 2.6% in 2012, and 4.9% in 2014. (*P* = 0.001).

### Baseline characteristics of IBD patients visiting the ED

A total of 190 cases of patients who visited ED were enrolled in the study (Table 1). One hundred and thirty-three (70.0%) were diagnosed with CD and 57 (30.0%) were diagnosed with UC. One hundred and twenty-four (65.3%) were males and the mean age was 40.0 years. Both age at ED presentation and age at diagnosis demonstrated a bimodal distribution. The median duration of illness was 37 months and the median time interval between the last AD and ED was 25 days.

**Table 1:** Patient demographics and baseline characteristics.

Variables	CD (N = 133)			UC (N = 57)			Total (N = 190)			*P* value
Age (years)	33.6	± 13.9	44.7		± 15.8			40.0	± 16.5	< 0.001
Age at diagnosis (years)	27.8	± 13.0	37.0		± 15.3			31.4	± 15.1	< 0.001
Sex–n (%)										0.464
Males	89	(66.9%)	35		(61.4%)			124	(65.3%)	
Disease duration (month)	38	(11, 109)	37		(18, 76)			37	(14, 96)	0.739
Comorbidity index–n (%)										0.160
≥1	2	(1.5%)	3		(5.6%)			5	(2.6%)	
Time interval between the last AD and ED (days)	23	(6, 44)	31		(9, 71)			25	(7, 48)	0.116
Medication exposure (%)										
5-ASA	129	(100%)	50		(98.0%)			179	(99.4%)	0.283
AZA/6-MP	71	(55.9%)	12		(25.5%)			83	(47.7%)	< 0.001
Corticosteroid	83	(64.8%)	28		(56.0%)			111	(62.4%)	0.274
Corticosteroid dose before ED visit										
>0, <20 mg/d	23	(18.1%)	9		(17.6%)			32	(18.0%)	
≥ 20 mg/d	16	(12.6%)	5		(9.8%)			21	(11.8%)	
Biologics	13	(10.2%)	3		(6.4%)			16	(9.2%)	0.563
Biologics now	12	(9.5%)	2		(4.3%)			14	(8.0%)	
Biologics before	1	(0.8%)	1		(2.1%)			2	(1.1%)	
Disease location (CD)										N/A
L1	48	(36.0%)	N/A					N/A		
L2	3	(2.3%)								
L3	74	(55.6%)								
Unknown	8	(6.0%)								
Disease behavior (CD)										N/A
B1	68	(51.1%)	N/A					N/A		
B2	28	(21.1%)								
B3	29	(21.8%)								
Unknown	8	(6.0%)								
History of CD-associated surgery–n (%)	55	(41.4%)	N/A					N/A		N/A
Montreal classification (UC)										
E1	N/A		11			(19.3%)		N/A		N/A
E2			18			(31.6%)				
E3			19			(33.3%)				
Unknown			9			(15.8%)				

*CD* Crohn’s disease, *UC* ulcerative colitis, *AD* ambulatory department, *ED* emergency department

Data regarding age and age at diagnosis are presented as mean ± standard deviation. Data for disease duration and time interval between the last AD and ED are presented as median (IQR).

Comparing CD patients with UC patients, CD patients were younger in age at ED presentation and at diagnosis, and more frequently received azathioprine or 6-mercaptopurine (6-MP) treatment. There were no significant differences in current or prior administration of 5-aminosalicylic acid (5-ASA), steroid, and biologics between UC and CD patients.

The disease location of CD was L1 (ileum) in 48 (36.0%), L2 (colon) in 3 (2.3%), L3 in 74 (55.6%), and unknown in 8 (6.0%) patients. The disease behavior of CD was B1 in 68 (51.1%), B2 in 28 (21.1%), B3 in 29 (21.8%), and unknown in 8 (6.0%) patients. The extent of UC was proctitis in 11 patients (19.3%), left-sided colitis in 18 (31.6%), extensive colitis in 19 (33.3%), and unknown in 9 (15.8%) patients.

### Clinical outcomes of IBD patients visiting the ED

The most common chief complaint of CD patients was abdominal pain (89 of 133 cases, 66.9%) while that of UC patients was hematochezia (24 of 57 cases, 42.1%) (Table 2). Abdominopelvic computed tomography (APCT) exam was performed more often in CD patients (61 of 129 cases, 46.6%) than in UC patients (15 of 57 cases, 26.3%) (*P* = 0.009). UC patients (27 of 57 cases, 47.4%) underwent more colonoscopy or sigmoidoscopy procedures than CD patients (9 of 133 cases, 6.9%) (*P* < 0.001). The hospitalization rate of CD patients was 46.6% (62 of 133 cases), while that of UC patients was 59.6% (34 of 57 cases) (*P* = 0.100). The median hospitalization duration for CD and UC patients was not different (10 days) (*P* = 0.295). A total of 5 (3.9%) CD patients underwent surgical intervention, as did 4 (7.0%) UC patients.

**Table 2:** Patient demographics and baseline characteristics.

Variables	CD (N = 133)			UC (N = 57)		Total (N = 190)			*P*-value
Chief complaint									<0.001
Abdominal pain	89	(66.9%)	12		(21.1%)		101	(53.2%)	
Hematochezia	9	(6.8%)	24		(42.1%)		33	(17.4%)	
Other GI symptom	9	(6.8%)	13		(22.8%)		22	(11.6%)	
Non-GI symptom	26	(19.5%)	8		(14.0%)		34	(17.9%)	
Abdomen CT*	61	(46.6%)	15		(26.3%)		76	(40.4%)	0.009
Colonoscopy/Sigmoidoscopy	9	(6.9%)	27		(47.4%)		36	(19.1%)	<0.001
Hospitalization	62	(46.6%)	34		(59.6%)		96	(50.5%)	0.100
Hospitalization duration (day)	10	(5, 14)	10		(6, 17)		10	(5, 17)	0.295
Surgery	5	(3.8%)	4**		(7.0%)		9	(4.8%)	0.351

*CD* Crohn’s disease, *UC* ulcerative colitis

All variables except hospitalization duration are reported as n (%). Data of hospitalization duration is presented as median (IQR).

*CT angiography (n = 2) in UC patents is included.

**Total colectomy (n = 2), duodenal perforation repair (n = 1), appendectomy (n = 1)

### Risk factors for hospitalization

In CD, patients who were male, with HR ≥ 100 bpm, with serum WBC > 10.0 x 10^3^/μL and CRP levels > 0.5 mg/dL, with stricturing (B2) or penetrating disease (B3), and had exposure to steroids were at higher risk of hospitalization by univariate logistic regression. In the multiple-variable regression analysis, penetrating disease (B3) (OR 3.54, *P* = 0.017) and presence of steroid exposure (OR 2.35, *P* = 0.047) remained independent risk factors for hospitalization (Table 3).

**Table 3:** Risk factors for hospitalization in Crohn’s disease.

Variables	Hospitalization	Univariable Analysis	Multivariable Analysis	
		*P-*value	OR (95% CI)	*P-*value
Sex				
Male	48/89 (53.9%)	0.018	2.183 (0.868, 5.491)	0.097
Female	14/44 (31.8%)		1.000 (reference)	
Disease Behavior		0.017		
B1	25/68 (36.8%)		1.000 (reference)	
B2	14/28 (50.0%)		1.582 (0.607, 4.123)	0.348
B3	20/29 (69.0%)		3.545 (1.256, 10.011)	0.017
Steroid exposure				
Yes	44/83 (53.0%)	0.099	2.352 (1.010, 5.480)	0.047
No	17/45 (37.7%)		1.000 (reference)	
HR				
<100 bpm	37/92 (40.2%)	0.027	1.000 (reference)	0.066
≥100 bpm	25/41 (61.0%)		2.222 (0.950, 5.198)	
Serum WBC				
≤10.0 x 10^3^/**μL**	22/64 (34.4%)	0.002	-	-
>10.0 x 10^3^/**μL**	61/127 (48.0%)			
Serum CRP				
≤0.5 mg/dL	11/32 (34.4%)	0.066	-	-
>0.5 mg/dL	50/94 (53.2%)			

*OR* odds ratio, *CI* confidence interval, *HR* heart rate, *WBC* white blood cell count, *CRP* C-reactive protein

All variables except hospitalization are reported as n (%).

In UC, a serum CRP level > 0.5 mg/dL was the only independent risk factor to predict hospitalization. Disease extent was not a risk factor for hospitalization (Table 4).

**Table 4:** Risk factors for hospitalization in ulcerative colitis.

Variables	Hospitalization	Univariable Analysis	Multivariable Analysis	
		*P-*value	OR (95% CI)	*P-*value
Sex				
Male	23/35 (65.7%)	0.239	-	-
Female	11/22 (50.0%)			
Disease extent				
Proctitis	6/11 (54.5%)			
Left-sided colitis	11/18 (61.1%)	0.859	-	-
Extensive colitis	12/19 (63.2%)			
Systolic BP				
>100 mmHg	29/52 (55.8%)	0.074	-	-
≤100 mmHg	5/5 (100.0%)			
Serum WBC				
≤10.0 x 10^3^/**μ**L	18/30 (60.0%)	0.761	-	-
>10.0 x 10^3^/**μ**L	16/25 (64.0%)			
Serum CRP				
≤1.0 mg/dL	11/26 (42.3%)	0.003	1.000 (Reference)	
>1.0 mg/dL	22/27 (81.5%)		5.400 (1.372, 21.260)	0.016

*OR* odds ratio, *CI* confidence interval, *BP* blood pressure, *WBC* white blood cell count, *CRP* C-reactive protein

All variables except hospitalization are reported as n (%).

### Risk factors for the length of hospitalization stay

**Fig 4:**
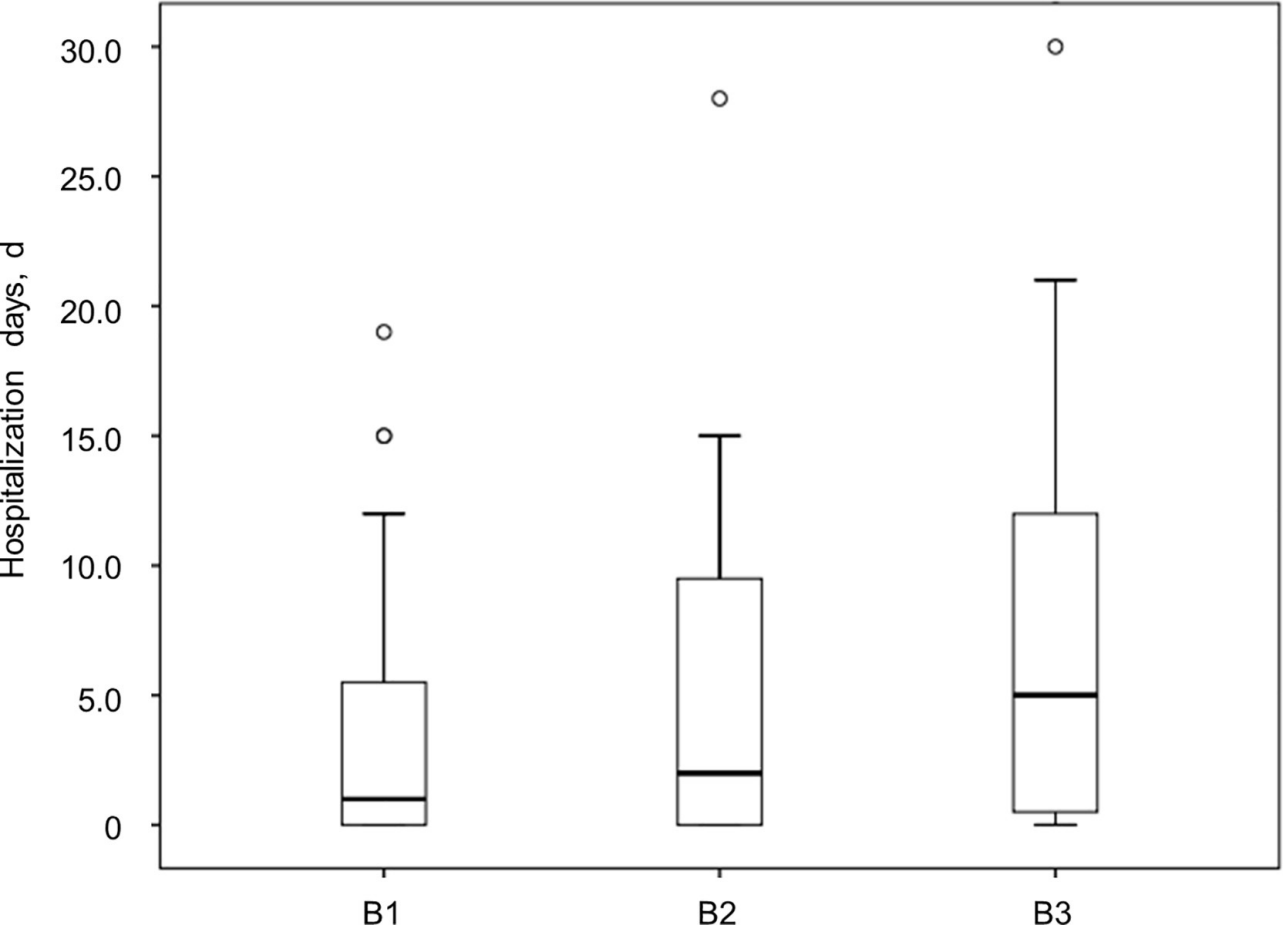
Length of hospitalization stay according to disease behavior in CD patients. The length of hospitalization stay was longer in patients with B3 than in patients with B1 or B2.

For CD, the length of hospitalization stay was longer in patients with penetrating disease (B3) than in patients with non-stricturing, non-penetrating disease (B1), or stricturing disease (B2). But the difference between them was not statistically significant. (B1 vs B2: *P* = 0.290; B2 vs B3: *P* = 0.211; B1 vs B3: *P* = 0.058) (Fig 4). For UC, hospitalization days in patients were not associated with the extent of disease.

## Discussion

In the current study, the absolute number of IBD-related visits increased but the proportion of ED visits decreased from the years 2007 to 2014. The proportion of IBD patients who had received or under treatment with biologics increased from 2007 to 2014. The increase in biologics use might be one of the major causes of the decreased proportion of ED visits by IBD patients. There were no significant differences in variables associated with severity (e.g. baseline characteristics, disease extent and behavior, and hospitalization rates) according to the year of ED visit.

In Asian countries, the disease location for CD and UC has been found to be different to those found in other studies from Western countries.[15] In these countries, CD has been found to involve small bowel, colon, and both small bowel and colon in equal proportions of patients. On the other hand, ileocolonic disease appeared has been reported as the predominant type in East Asia.[16] A population-based study in a district of Seoul, the capital of South Korea, reported that among 138 CD patients, ileocolic disease (L3) at diagnosis were 66.7%, while isolated small bowel disease (L1) and isolated colonic disease (L2) were only 21.0% and 12.3%, respectively.[17] In the current study, the disease location of CD patients who visited the ED was L1 in 46 (35.7%), L2 (colon) in 3 (2.3%) and L3 in 72 (55.8%). Compared with the previous population-based study in South Korea, the proportion of L1 was relatively high, while that of L2 was low, which means unexpected exacerbations or complications might occur more frequently in patients with isolated small bowel disease, resulting in more ED visits. Unlike UC, the main symptom in colitic CD patients is usually diarrhea, rather than bloody stool, which explains the lower frequency of ED visits by colitic CD patients. The results of a previous study indicated that the number of patients with isolated colonic disease had not increased significantly from 1986 to 2005, while there had been steady rise in the numbers with isolated small bowel disease and both small bowel and colonic disease.[17] It must be emphasized that clinicians should pay greater attention to patients presenting with L1 disease at the AD. In the present study, we could not evaluate the differences in baseline characteristics between patients with L1 and L2 disease since the number of patients with L2 was low. When compared to L3 patients, those with L1 disease have less proportion of penetrating disease (B3).

Only a few studies have analyzed predictors of hospitalization at the ED in IBD patients.[18, 19] Neither study reported a multivariable analysis of predictors of hospitalization in subgroups of CD or UC patients. A population-based study in Canada reported that having been prescribed corticosteroids at least twice within the previous year was a significant predictor of hospitalization in IBD patients.[18] Another study based on a U.S.A. nationwide all-payer ED database with ICD codes reported that intra-abdominal abscess, fever, and abnormal white cell count were factors associated with hospitalization.[19] These results are mostly consistent with our findings.

In the present study, disease behavior and the presence of steroid exposure were significantly associated with hospitalization in CD. This might not be surprising, as disease behavior according to the Montreal classification is based on the presence of intestinal complications such as stricture or fistula. However, there have not been any studies evaluating the association between disease behavior before the ED visit and hospitalization in CD patients. Patients with steroid exposure were more likely to have had refractory disease, which explains the higher risk of hospitalization in CD. Since hospitalization risk does not depend on the laboratory and imaging findings performed after the ED visit, but on disease behavior and steroid exposure, the importance of accurate history taking cannot be more strongly stressed. CD patients with penetrating behavior or steroid exposure need to be examined thoroughly and hospitalization should be considered from the initial assessment and planning.

Serum CRP was the only factor to predict hospitalization in UC. Unlike CD patients, disease extent and presence of steroid exposure were not associated with hospitalization in UC. Progression and regression of disease extent are known to be more common in UC than in CD.[20] For these reasons, previous disease extent might not be useful in predicting hospitalization in UC. However, the proportion of E1 in UC patients who visited the ED was 19.3% in our study, which is much lower than the 41.2% reported in a population-based study in South Korea [17] and the 44.1% in a tertiary teaching hospital-based study in South Korea.[21] Therefore, patients with previous disease extent E1 in UC seems to have a lower probability of visiting ED than patients with more extensive disease. Even though, because previous disease extent is not associated with risk of hospitalization in UC patients who visit ED, we should carefully evaluate patients with ulcerative proctitis just like patients with more extensive colitis.

There are several limitations to this study. First, it is based on data from a single institution and retrospective design, so the possibility of selection bias should be considered. Electronic medical records and prescribed medications were reviewed thoroughly, but some clinical data, including medication history (i.e., administration of non-steroidal anti-inflammatory drugs [NSAIDs] or opioids) or activity index scores, could not be collected completely. These unmeasured data could possibly lead to confounding of the results. Second, the study population includes heterogeneous patients with different causes of ED visits.

In conclusion, the absolute number of IBD-related ED visits increased from 2007 to 2014, but the proportion of ED visits decreased from 2007 to 2014; there was no significant change in hospitalization rates. Disease behavior and presence of steroid exposure were associated with hospitalization among CD patients who visited the ED and elevated CRP was associated with hospitalization among UC patients.
