# Correction: Trends in emergency department visits and hospitalization rates for inflammatory bowel disease in the era of biologics

**DOI:** 10.1371/journal.pone.0216768

**Published:** 2019-05-07

**Authors:** Gunn Huh, Hyuk Yoon, Yoon Jin Choi, Cheol Min Shin, Young Soo Park, Nayoung Kim, Dong Ho Lee, Joo Sung Kim

There are errors in the Results subsection of the Abstract. The correct Results subsection is:

The proportion of IBD patients who visited ED was 11.9% in 2007, 9.2% in 2009, 8.1% in 2012, and 6.3% in 2014 (*P* = 0.002). The most common chief complaints were abdominal pain (66.9%) in Crohn’s disease (CD) patients and hematochezia (42.1%) in ulcerative colitis (UC) patients. The hospitalization rate following ED visits was 46.6% in CD patients and 59.6% in UC patients (*P* = 0.100). Multiple-variable analysis showed that significant risk factors associated with hospitalization in CD were aggressive disease behavior (odds ratio[OR] 3.54, *P* = 0.017) and presence of steroid exposure (OR 2.35, *P* = 0.047). Elevated C-reactive protein (CRP) (>0.5 mg/dL) (OR 5.40, *P* = 0.016) was the only risk factor associated with hospitalization in UC.

In the Trends in ED visits and hospitalization rates subsection of the Results, there are errors in the sixth, seventh, and eighth sentences. The correct sentences are:

There was no significant linear trend for hospitalization rates of ED patients (*P* = 0.587) (Fig 2). Hospitalization rate of IBD patients following ED was 65.2% in 2007, 42.4% in 2009, 47.8% in 2012, and 50.8% in 2014. In CD patients, hospitalization rate was 57.1% in 2007, 41.2% in 2009, 44.0% in 2012, and 50.0% in 2014 (*P* = 0.921). In UC patients, hospitalization rate was 77.8% in 2007, 43.8% in 2009, 57.9% in 2012, and 52.6% in 2014. (*P* = 0.585).

In the Clinical outcomes of IBD patients visiting the ED subsection of the Results, there is an error in the final sentence. The correct final sentence is:

A total of 5 (3.8%) CD patients underwent surgical intervention, as did 4 (7.0%) UC patients.

There is an error in the caption for of Fig 2, Hospitalization rates of patients with IBD (A), CD (B), and UC (C) visiting ED stratified by years. The *P* Value was not 0.610, but 0.587. Please see the complete, correct Fig 2 here.

**Fig 2:**
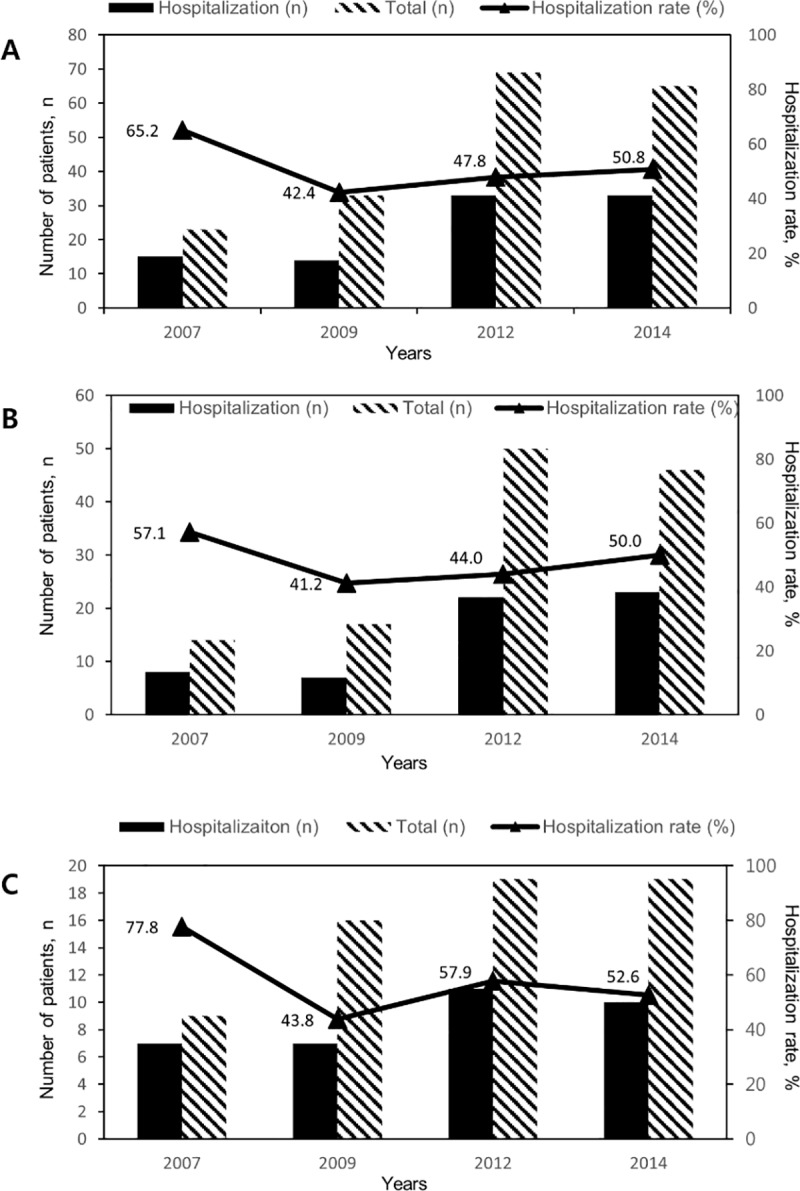
**Hospitalization rates of patients with IBD (A), CD (B), and UC (C) visiting ED stratified by years.** There was no significant linear trend for hospitalization rates of ED patients (*P* = 0.587).

There is an error in panel C of Fig 3, The number and proportion of patients with IBD (A), CD (B), and UC (C) treated with biologics stratified by years. The correct proportion of UC patients treated with biologics in 2014 was not 4.0%, but 4.9%. Please see the complete, correct Fig 3 here.

**Fig 3:**
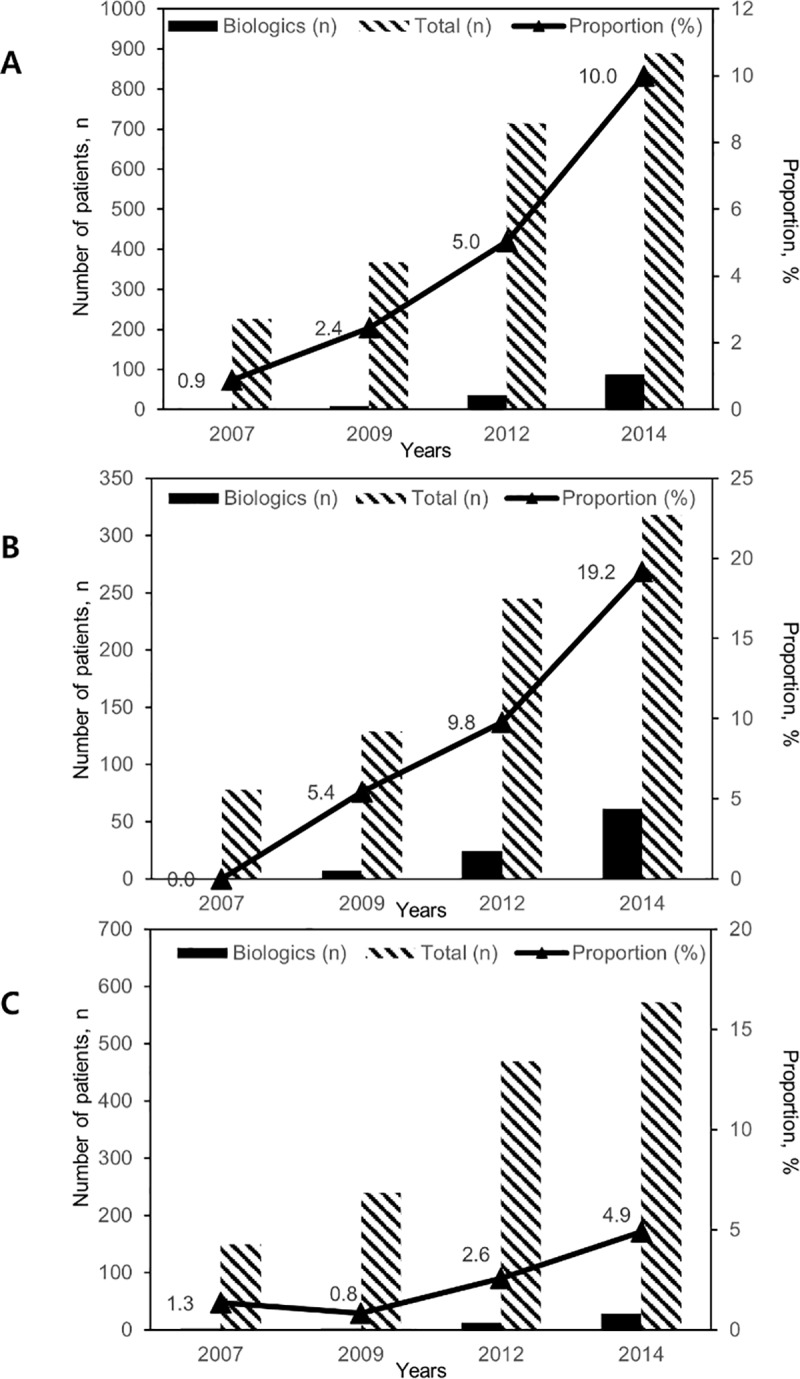
**The number and proportion of patients with IBD (A), CD (B), and UC (C) treated with biologics stratified by years.** The number and proportion of IBD patients who had received or under treatment with biologics increased from 2007 to 2014 (*P* <0.001).

There is an error in the last two rows of the first column of Table 4. The threshold for elevated serum CRP was not 1.0 mg/dL, but 0.5 mg/DL. Please see the complete, correct Table 4 here.

**Table 4:** Risk factors for hospitalization in ulcerative colitis.

Variables	Hospitalization	Univariable Analysis	Multivariable Analysis	
		*P-*value	OR (95% CI)	*P-*value
Sex Male Female	23/35 (65.7%) 11/22 (50.0%)	0.239	-	-
Disease extent Proctitis Left-sided colitis Extensive colitis	6/11 (54.5%) 11/18 (61.1%) 12/19 (63.2%)	0.859	-	-
			-	-
Systolic BP > 100 mmHg ≤ 100 mmHg	29/52 (55.8%) 5/5 (100.0%)	0.074	-	-
Serum WBC ≤ 10.0 x 10/**μ**L > 10.0 x 10/**μ**L	18/30 (60.0%) 16/25 (64.0%)	0.761	-	-
Serum CRP ≤ 0.5 mg/dL > 0.5 mg/dL	11/26 (42.3%) 22/27 (81.5%)	0.003	5.400 (1.372, 21.260)	0.016
